# Retraction notice to Diagnosis of Microvascular Angina Using Cardiac Magnetic Resonance: J Am Coll Cardiol 71 (2018) 969-979

**DOI:** 10.1016/j.jacc.2020.08.039

**Published:** 2020-10-20

**Authors:** Alexander Liu, Rohan S. Wijesurendra, Joanna M. Liu, John C. Forfar, Keith M. Channon, Michael Jerosch-Herold, Stefan K. Piechnik, Stefan Neubauer, Rajesh K. Kharbanda, Vanessa M. Ferreira

**Affiliations:** Oxford Centre for Clinical Magnetic Resonance Research, Division of Cardiovascular Medicine, Radcliffe Department of Medicine, University of Oxford, Oxford, United Kingdom; Oxford Heart Centre, John Radcliffe Hospital, Oxford, United Kingdom; Division of Cardiovascular Medicine, Radcliffe Department of Medicine, University of Oxford, Oxford, United Kingdom; Brigham and Women’s Hospital, Radiology, Cardiovascular Imaging, Boston, Massachusetts

This article has been retracted: please see Elsevier Policy on Article Withdrawal (https://www.elsevier.com/about/our-business/policies/article-withdrawl).

The *JACC* Journals Ethics Board has voted to retract this paper, relying on the findings of misconduct after an investigation by the University of Oxford (outlined below). The decision to retract the paper follows the conclusion of an investigation under the University of Oxford’s (“the University’s”) Code of Practice and Procedure on Academic Integrity in Research (“the Code”). The Registrar of the University convened a Panel under the Code. The Panel considered a number of issues, including in relation to this paper. The Panel concluded that the first author, Dr Alexander Liu, was responsible for misconduct in research. The Panel’s findings with regards to misconduct were limited to the actions of the first author. No other co-author was found to be involved in the misconduct. It is understood that the first author disagrees with the Panel’s findings. The first author has raised a complaint with the Office of the Independent Adjudicator (OIA) (The OIA reviews complaints from students about their higher education provider).

In relation to this paper, the Panel’s findings included that:

• rather than 50 individual patients in the study (28 CAD providing 35 vessels and 22 NOCAD providing 66), there were actually 26 CAD patients, providing 31 vessels and 25 NOCAD patients providing 60 vessels, but three patients each providing two vessels were double counted, providing 66 vessels in all. Only six patients met the stated definition of NOCAD;
• 5 controls with discrepant ages were included in the study;
• controls had been iteratively excluded from the analysis as the paper was revised, without explanation;
• that subjects used as controls in the study were patient volunteers and not healthy controls; and
• Figure 5 had been fabricated.

The Panel’s view was that this paper would likely need to be retracted from the literature as it had major irregularities and its conclusions were unsafe.

The following co-authors agree that a retraction is appropriate: Vanessa Ferreira, Rajesh Kharbanda, Stefan Neubauer, Stefan Piechnik, Michael Jerosch-Herold, Keith Channon, John C. Forfar, Joanna Liu and Rohan Wijesurendra.

